# CIBER-CLAP (CIBERCV Cardioprotection Large Animal Platform): A multicenter preclinical network for testing reproducibility in cardiovascular interventions

**DOI:** 10.1038/s41598-019-56613-6

**Published:** 2019-12-30

**Authors:** Xavier Rossello, Antonio Rodriguez-Sinovas, Gemma Vilahur, Verónica Crisóstomo, Inmaculada Jorge, Carlos Zaragoza, José L. Zamorano, Javier Bermejo, Antonio Ordoñez, Lisardo Boscá, Jesús Vázquez, Lina Badimón, Francisco M. Sánchez-Margallo, Francisco Fernández-Avilés, David Garcia-Dorado, Borja Ibanez

**Affiliations:** 1 Centro de Investigación Biomédica en Red en Enfermedades Cardiovasculares (CIBERCV), Madrid, Spain; 2 Centro Nacional de Investigaciones Cardiovasculares (CNIC), Madrid, Spain; 3 Laboratorio de Investigación en Enfermedades Cardiovasculares, Vall d’Hebron Institut de Recerca, Barcelona, Spain; 4 Programa ICCC-Institut de Recerca de l’Hospital de la Santa Creu i Sant Pau, IIB-Sant Pau, Barcelona, Spain; 5 Centro de Cirugía de Mínima Invasión Jesús Usón, Cáceres, Spain; 6 Servicio de Cardiologia, Hospital Universitario Ramón y Cajal, Instituto Ramón y Cajal de Investigación Sanitaria IRYCIS, Madrid, Spain; 7 Universidad Francisco de Vitoria, Madrid, Spain; 8 Department of Cardiology, Hospital General Universitario Gregorio Marañón, Instituto de Investigación Sanitaria Gregorio Marañón and Facultad de Medicina, Universidad Complutense de Madrid, Madrid, Spain; 9 Instituto de Biomedicina de Sevilla, Sevilla, Spain; 10 Instituto de Investigaciones Biomédicas Alberto Sols (Centro Mixto CSIC-UAM), Madrid, Spain; 11 Cardiology Department, IIS-Fundación Jiménez Díaz Hospital, Madrid, Spain

**Keywords:** Biological techniques, Interventional cardiology

## Abstract

Despite many cardioprotective interventions have shown to protect the heart against ischemia/reperfusion injury in the experimental setting, only few of them have succeeded in translating their findings into positive proof-of-concept clinical trials. Controversial and inconsistent experimental and clinical evidence supports the urgency of a disruptive paradigm shift for testing cardioprotective therapies. There is a need to evaluate experimental reproducibility before stepping into the clinical arena. The CIBERCV (acronym for Spanish network-center for cardiovascular biomedical research) has set up the “Cardioprotection Large Animal Platform” (CIBER-CLAP) to perform experimental studies testing the efficacy and reproducibility of promising cardioprotective interventions based on a pre-specified design and protocols, randomization, blinding assessment and other robust methodological features. Our first randomized, control-group, open-label blinded endpoint experimental trial assessing local ischemic preconditioning (IPC) in a pig model of acute myocardial infarction (n = 87) will be carried out in three separate sets of experiments performed in parallel by three laboratories. Each set aims to assess: (A) CMR-based outcomes; (B) histopathological-based outcomes; and (C) protein-based outcomes. Three core labs will assess outcomes in a blinded fashion (CMR imaging, histopathology and proteomics) and 2 methodological core labs will conduct the randomization and statistical analysis.

## Introduction

After acute myocardial infarction (AMI), early myocardial reperfusion is the most effective strategy for reducing myocardial infarct size (IS) and potentially improving clinical outcomes^1,2^. However, the process of restoring blood flow to the ischemic myocardium can paradoxically induce additional injury to the tissue. This phenomenon, known as ischemia/reperfusion injury (IRI), can therefore limit the beneficial effects of myocardial reperfusion^3–7^. Unlike the successful implementation of reperfusion therapy into the clinical setting^3,8–10^, the translation of novel cardioprotective interventions aimed to protect the heart against IRI has mostly resulted in overall disappointing results^11–16^. It is recognized that protecting the ischemic myocardium remains one of the top ten unmet clinical needs in cardiovascular medicine^17^.

## Cardioprotection: Reasons for Loss in Translation

Despite many cardioprotective interventions have shown to protect the heart against IRI in the experimental setting^3,12^, only few of them have succeeded in translating their findings into positive proof-of-concept clinical trials demonstrating either reduction in myocardial IS, or improvement in left ventricular systolic function^18–22^. The reasons for this disappointing translation have been broadly discussed elsewhere and are a matter of intense debate^1,12,23^. Some of these reasons have been attributed to methodological flaws and lack of scientific rigor, but the underlying issue is the lack of reproducibility. Critical methodological issues, such as blinding, randomization, a priori sample size estimation or well-designed prespecified protocols are often neglected in the experimental setting, leading to an overoptimistic (and sometimes biased) interpretation of the findings^24^. Overall, the replication and reproducibility of experimental results across laboratories are so alarmingly low^25,26^ that several Research Working Groups have made their recommendation to address this issue^27–29^. Moreover, the unmeasured bias towards publication of positive results (neutral or negative preclinical results are barely reported^30–32^) does not help to have a clear-cut idea of which therapies depict consistent results.

### Previous attempts to assess variability across laboratories

There have been few attempts to assess the consistency and reproducibility of treatment effects across different labs in the field of cardioprotection. In one of them, well-recognized research groups tested the impact of the adenosine A1 receptor agonist GR79236 on myocardial IS when administered at reperfusion onset. In a three-center experimental study, the investigators designed a blinded, randomized treatment protocol in sixty rabbits^33^. Although there were some small local differences in anesthetic regimens, AMI protocol and IS determination, the overall study performance was relatively homogenous across laboratories^33^. Fifteen years later, the NHLBI-Sponsored Consortium for preclinicAl assESsment of cARdioprotective Therapies (CAESAR)^24,34^ initiative assessed the cardioprotective effect provided by local ischemic preconditioning (IPC) in 3 different species (mouse, rabbit and pig) at 3 different institutions of the consortium (2 centers/species). Beyond reporting a consistent IS-sparing effect across centers, they also observed a species-related effect size gradient (IS reduction was largest in mice, intermediate in rabbits and lower in pigs). Interestingly, Jones *et al*. acknowledged that 2 other cardioprotective interventions (sodium nitrate and sildenafil citrated) were also tested through the CAESAR platform, but failed to demonstrate a consistent effect of limiting IS^34^. While these two previous attempts have been the first step to evaluate effect size consistency, the reality is that we still lack an active platform to thoroughly assess cardioprotective therapies before stepping in into proof-of-concept clinical trials. There is a need to make comprehensive evaluations of cardioprotective therapies, from both an effective perspective (assessing myocardial IS through imaging and histological techniques), and a mechanistic perspective (assessing known and unknown molecular pathways).

### The CIBER-CLAP platform

The CIBERCV (acronym for Spanish network-center for cardiovascular biomedical research Spanish, “Centro de Investigación Biomédica en Red en enfermedades CardioVasculares” —in Spanish) has designed the Cardioprotection Large Animal Platform” (CIBER-CLAP) platform as part of its program “arterial disease, myocardial ischemia. and structural damage” and research line “Myocardial ischemia and reperfusion” from the CIBERCV. The CIBER-CLAP platform is inspired by the same principles than trialists use to conduct randomized clinical trials (RCTs). The ultimate aim is to perform experimental studies testing the efficacy of promising cardioprotective interventions based on a pre-specified design and protocols, randomization, blinding assessment and other robust methodological features, as well as to respect some key reporting-style aspects in the relevant publications^35^. The essence is to test reproducibility across research centers. If one therapy is not reproducible in the experimental setting, it is unlikely that it will be effective in the more complex clinical setting, where a larger number of confounding factors have an impact on the results^36^. Nevertheless, it has to be acknowledged that reproducibility does not guarantee translatability. Although the first step is to have robust cardioprotective therapies, uncontrolled clinical variations, such as the duration of ischemic, infarct location, comorbid conditions and other clinical features^11,12^ may play a role in failing to translate a highly reproducible therapy.The CIBER-CLAP platform is intended to be used for the rigorous evaluation of candidate cardioprotective interventions. However, before being used to screen IS-limiting therapies, it is necessary to validate the platform by testing its ability to: (1) produce comparable and reproducible results across 3 different centers using a single protocol in the same translational model and the same blinded core-labs to evaluate endpoints; (2) detect a consistent cardioprotective effect not only in different experimental outcomes, such as myocardial IS reduction measured by cardiac magnetic resonance (CMR) and histology, but also on the regulation of pro-survival signaling pathways (measured by antibody-based approaches and proteomics).

This study aims to establish the CIBER-CLAP platform; hence in this first pilot investigation, we are interested in assessing reproducibility across laboratories rather than testing a therapy in itself (i.e., test the network, not the therapy, which is known to be effective). To build up this highly coordinated network, protocols have been agreed among researchers in discussions that took place during approximately one year. It was also agreed that the study design, power calculation, randomization, and analyses would be done centrally, as well as the evaluation of the experimental outcomes. We aim to control the potential “researcher bias” by performing the experiments in several institutions and establishing a central adjudication of the outcomes, but allowing for certain variability (i.e. source of the animals, husbandry conditions, water source, veterinary staff…) to provide some external validity.

### Rationale for using local ischemic preconditioning (IPC)

The IPC phenomenon designates a intervention by which the myocardium can endogenously be protected from lethal IRI through the application of several cycles of transient ischemia and reperfusion performed before the index sustained ischemic insult. IPC was firstly described in a dog model of AMI by Murry *et al*.^37^ and has been subsequently replicated in multiple pre-clinical models^38,39^ as well as in other organs^40^. Moreover, it has also been proved to take place in humans^41,42^. Importantly, the concept of IPC has evolved into “ischemic conditioning”^43^, a broader term that encompasses a number of similar endogenous cardioprotective therapies, applied either to the heart (ischemic preconditioning and postconditioning^44^) or to a distant organ (remote ischemic pre-^45^, per-^20^ or post-conditioning^46,47^). Although the translational potential of local IPC is inevitably limited by the fact that the intervention needs to be applied before the index ischemia, which is unpredictable in AMI patients, this phenomenon has become pivotal for the study of other cardioprotective therapies for several reasons. First, it was the first therapy to demonstrate a reduction in IS through an endogenous mechanism, thus helping to identify molecular targets amenable to pharmacological manipulation. Second, IPC is thought to have an impact not only on the ischemic damage, but also on its reperfusion-related injury. The manipulation of three signaling pathways (RISK, SAFE and PKG/eNOS) at the onset of reperfusion^48–50^ has a huge translational value^48^, given that this underlying molecular signaling can be mimicked by pharmacological agents that may be administered either during ongoing ischemia or immediately during reperfusion (ie. in the ambulance or the cath lab)^9,51,52^. It has been already demonstrated that these pathways are not only recruited by IPC, but also by other pharmacological agents^53^, making this underlying molecular architecture a shared universal pathway for many cardioprotective therapies. Third, IPC has been a subject of intense research, resulting in over 10,000 publications in the last three decades^39^ and has become the paradigm of cardioprotection. In fact, IPC is the most powerful and reproducible cardioprotective intervention to date^54^. It is commonly used as a positive control when testing novel cardioprotective therapies^30,48,53^. The ultimate reason for using IPC as the first strategy in CIBER-CLAP is to use a therapy without controversy on its ability to limit IS and thus to remove one relevant confounding factor from the evaluation of the initiative. Since the goal of this study is not to test the efficacy of a given therapy, rather to test the performance of the network in terms of reproducibility and logistics, IPC represents the ideal first step. Table 1 summarizes the rationale for using local IPC as the pivotal intervention for testing the performance of the CIBER-CLAP platform for future applications.

**Table 1:** Rationale for using local IPC in CIBER-CLAP.

1	IPC is considered the most robust and reproducible intervention available to protect the heart against myocardial IRI, making it ideal for assessing effect size variability across laboratories
2	IPC is considered the cornerstone of cardioprotection after more than three decades of research^39^. It is, therefore, a well-known and fully recognized therapy
3	The pro-survival signaling pathways underlying the cardioprotective effect provided by IPC are relatively well understood^50,78^: the RISK pathway (comprising PI3K-Akt and MEK1/2-ERK1/2)^79,80^, the SAFE pathways (comprising TNFα and JAK-STAT3)^81,82^ and the PKG/eNOS signaling cascade^83^. The high consensus over the role of these pathways is crucial to perform mechanistic studies across labs within the CIBER-CLAP platform.
4	The underlying mechanism provided by IPC has become the paradigm for cardioprotection [23]: its prosurvival pathways must be activated at the time of early reperfusion for IPC to provide protection^48^ and, importantly, they can be recruited by other pharmacological agents such insulin^53^, bradykinin^84^ or statins^85^. It has been demonstrated that most cardioprotective therapies share these common pathways, which are considered, making universal the underlying signaling cascade^48,86^
5	IPC is a phenomenon occurring in humans and thus translatable. It has been shown protective effects either in the theatre^41^ or in the human atrial trabeculae model^42^. It has also been described to provide an increased exercise tolerance following an episode of angina (“Warm-up angina”)^87^ and resulting in smaller IS and improved clinical outcomes when presented before an AMI (“Pre-infarct angina”)^88^
6	The ultimate reason for using IPC as the first strategy in CIBER-CLAP is to use a therapy without controversy on its ability to limit IS and thus to remove one relevant confounding factor from the evaluation of the initiative. Since the goal of this study is not to test the efficacy of a given therapy, rather to test the performance of the network in terms of reproducibility and logistics, IPC represents the ideal first step.

AMI, acute myocardial infarction; IPC; ischemic preconditioning; IRI, ischemia/reperfusion injury; RISK, Reperfusion Injury Salvage Kinase; SAFE. Survivor Activator Factor Enhancement.

## Study Design

The first study to be conducted through the CIBER-CLAP is a multicenter randomized controlled open-label blinded endpoint experimental trial testing the application of IPC in a pig model of AMI. All animal studies have been approved by the Comunidad de Madrid ethics committee, and CNIC’s institutional review board. All animal procedures will conform to EU Directive 2010/63EU and Recommendation 2007/526/EC regarding the protection of animals used for experimental and other scientific purposes.

### Study hypothesis and end points

The main hypothesis of the CIBER-CLAP study is that the application of local IPC has consistent cardioprotective effects across three different centers that use a common protocol. This will be the basis for having a solid control in subsequent hypothesis testing further cardioprotective therapies. This hypothesis is relevant because even within the same laboratory, IPC experiments performed by the same researcher have shown some discrepancies (i.e. mean difference in IS between IPC and control ranging between −12.3 and – 38.1)^54^, and is novel because molecular pathways have been barely evaluated and have shown inconsistent findings in the swine model^1,55^.

The primary endpoint is the observation of a consistent reduction on myocardial IS as measured by CMR across laboratories. The secondary endpoints are: 1) to observe a consistent reduction on myocardial IS as measured by histological techniques across centers; 2) to detect the consistent phosphorylation of Akt, ERK and Stat3 in the IPC arm in comparison to the control arm; 3) to detect a consistent pattern of alterations in the plasma and heart proteomes indicative of the activation of signaling processes related to IPC and the inhibition of protein pathways and modifications known to reflect IRI damage. Note that the last two endpoints will also be very useful to establish robust protocols to evaluate molecular changes associated to IPC, which could be used in future uses of the CIBERCLAP. Besides, proteomics will offer a wide evaluation of a large range of biological processes, allowing detection of minor molecular changes that would otherwise pass unnoticed.

### Parallel subsets of experiments

**Figure 1:**
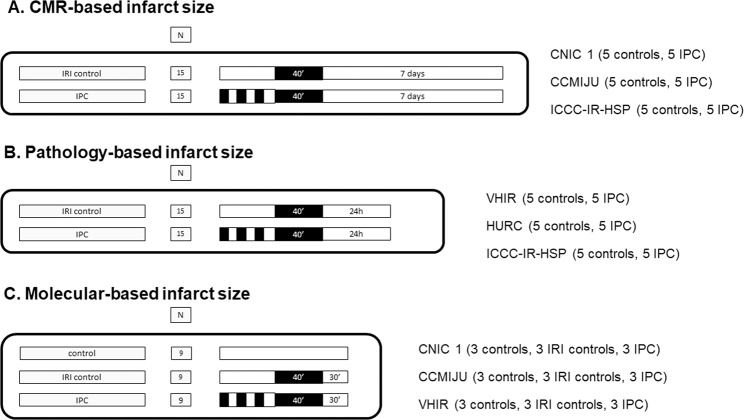
Study design involving at least 3 experimental groups per outcome and 5 core laboratories.

The study population will consist of 87 farm pigs between 25 kg to 38 kg at the time of infarct induction. We designed a randomized, control-group, open-label blinded endpoint experimental trial assessing a single intervention (IPC) in three separate sets of experiments. Each set of experiments will be performed in parallel by three laboratories and each of them will be aimed to study one type of outcome: A) CMR-based outcomes; B) histopathological-based outcomes; and C) protein-based outcomes. There will be 5 core labs: 3 assessing outcomes in a blinded fashion (CMR imaging, histopathological evaluation and proteomics) and 2 methodological core labs (randomization and statistical analysis). Although we could set up a core lab for Western Blotting procedures, we preferred to also assess reproducibility using three different centers. The number of pigs allocated per group and the summary of the outcomes are presented in Table 2. The study design and the involvement of each center within the organigram are summarized in Fig. 1.

**Table 2:** Outcomes to be assessed by sets of experiments.

Subset	Type of outcomes (n)	Outcomes
A	CMR-based (n = 30)	**Myocardial infarct size (%LV);** LVEF (%); edema extension (% LV); intramyocardial hemorrhage (presence and extension); microvascular obstruction (presence and extension).
B	Histopathological-based (n = 30)	**Myocardial infarct size (%LV);** AAR (% LV area) Myocardial infarct size (%AAR);
C	Western blot-based (n = 27)	**Phosphorylation of Akt, ERK and Stat3 relative to total protein**
C	Proteomics	**Exploratory analysis**

In bold, the primary endpoint for each subset of experiments.

AAR, area at risk; CMR, cardiac magnetic resonance; LV, left ventricular.

The three sets of experiments will have a common reperfused AMI protocol (closed-chest 40-min left anterior descending (LAD) coronary artery occlusion followed by reperfusion) and intervention (three 5-minute cycles of IPC). They differ in their follow-up according to the nature of the outcome assessments: in set A, pigs will undergo CMR scans at baseline and on day 7 post-procedure, when they will be euthanized; in set B, pigs will undergo 24 h reperfusion and then euthanized to perform the pathology assessment; and in set C, pigs will undergo 30 min reperfusion and myocardial tissue samples will be then collected from the ischemic, border and remote areas to evaluate kinase phosphorylation through Western blot techniques. Samples will be also be collected from the ischemic and remote areas for proteomics evaluation.

### Experimental protocols

**Figure 2:**
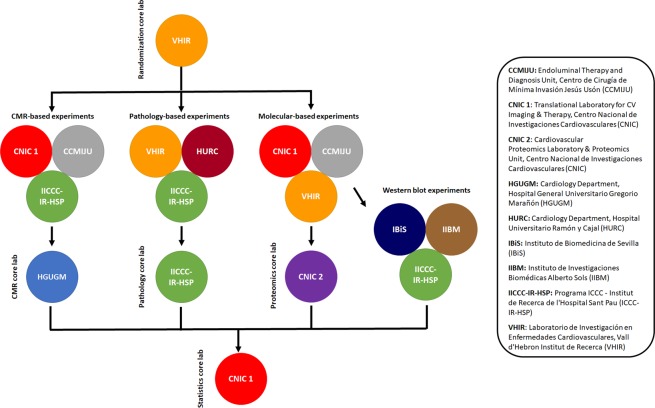
Experimental protocols by outcomes and centers. CCMIJU, Centro de Cirugía de Mínima Invasión Jesús Usón; CNIC 1, Centro Nacional de Investigaciones Cardiovasculares (CNIC); HURC, Hospital Universitario Ramón y Cajal (HURC); ICCC-IR-HSP, Programa ICCC - Institut de Recerca de l’Hospital Sant Pau (ICCC-IR-HSP); VHIR, Vall d’Hebron Institut de Recerca (VHIR).

A study protocol flowchart is shown in Fig. 2.

#### Pig model of acute myocardial infarction

The pig model of acute myocardial infarction was chosen because it closely represents the clinical setting due to its anatomical and physiological similarities to the human heart, and because large animals are the obligatory step before initiating human trials^3,56,57^. They have also shown to be a very reliable experimental model^3,58^.

The IRI protocol has been detailed elsewhere^31,58^, though some variations have been implemented to homogenize the protocol across centers. To avoid the protective effects exerted by volatile anesthetics, anesthesia will be induced by intramuscular injection of ketamine (20 mg/kg), xylazine (2 mg/kg), and midazolam (0.5 mg/kg) and maintained by continuous intravenous infusion of ketamine (3 mg/kg/h), xylazine (0.45 mg/kg/h), and midazolam (0.4 mg/kg/h). Animals will be intubated and mechanically ventilated with a 28% fraction of inspired oxygen. A single bolus of 300 IU/kg unfractionated heparin will be administered at the onset of the procedure as soon as the femoral central venous sheath is inserted. The LAD coronary artery will be occluded immediately distal to the origin of the first diagonal branch for 40 min with an angioplasty balloon introduced via the percutaneous femoral route using the Seldinger technique. Balloon location, maintenance of inflation and post-reperfusion patency will be monitored angiographically. To prevent malignant ventricular arrhythmias, a continuous infusion of amiodarone (300 mg/h) will be initiated at the time of balloon occlusion (no bolus) and maintained until 30-min post-reperfusion. In cases of ventricular fibrillation, non-synchronized shocks will be delivered using a biphasic defibrillator at 200 Joules.

#### Intervention

Prior to the prolonged index LAD occlusion, IPC will be induced by three 5-minute cycles of balloon inflation/deflation at the same level of the subsequent LAD occlusion. This protocol was chosen based on our previous successful experience^59^ as well as on previous evidence suggesting that IPC is a steady graded phenomenon conferring summative protection by supplementary cycles^60^, with an upper limit where an excessive number of cycles can be associated with loss of protection^61^.

#### Plasma collection

Plasma collection (2 mL) will take place through the marginal vein of the ear at baseline (sets of experiments A, B and C), 30 min post-reperfusion (sets A, B and C), 24 h post-AMI (set B) and 7 days post-AMI (set A).

#### CMR protocol

CMR studies will be performed at baseline (within 24 h from AMI induction) and day 7 post-AMI to assess myocardial IS (% of LV), LV function and other CMR-based outcomes summarized in Table 2. Seven days is chosen based on solid preclinical and clinical experience suggesting it is the ideal time point for the evaluation of irreversible damage after IRI^58,59,62,63^. The baseline CMR does not include late gadolinium enhancement imaging. On each CMR exam, pigs will be anesthetized by intramuscular injection of ketamine, xylazine, and midazolam and maintained by continuous intravenous infusion of the same drugs, as described above.

All studies will be performed in a Philips 1.5 or 3-T Achieva Tx whole-body scanner (Philips Healthcare, Best, the Netherlands) equipped with a 32-element phased-array cardiac coil. Segmented cine steady-state free precession (SSFP) will be performed to acquire 13–15 contiguous short-axis slices covering the heart from the base to the apex to assess LV mass, LV volumes, and ejection fraction with the following parameters: FOV of 280 × 280 mm; slice thickness of 6 mm without gap; minimum TR and TE for each scanner, flip angle 60° for 1.5-T and 45° for 3-T; cardiac phases 30; voxel size 1.6 × 1.6 mm. Edema imaging will be performed using a T2-weighted, triple inversion-recovery fast spin-echo (T2W-STIR) sequence with the following parameters: FOV of 280 × 280; 15 short-axis slices with thickness of 6 mm and no gap; TR 2 heartbeats; TE 80 ms; voxel size 1.6 × 1.6 mm; STIR delay 160 ms or 210 ms in 1.5-T and 3-T, respectively; echo train length 16; and 2 NEX. To determine myocardial IS, late gadolinium enhancement imaging will be performed 10–15 min after the administration of 0.2 mmol/kg gadopentate dimeglumine using a T1 inversion-recovery turbo field echo (T1-IR-TFE) sequence with the following parameters: FOV of 280 × 280 mm; 13–15 short-axis slices with a thickness of 6 mm and no gap; TR 5.6 ms; TE 2.8 ms; voxel size 1.6 × 1.6 mm; inversion time will be optimized to null normal myocardium; and 2 NEX.

#### CMR data analysis

All CMR images will be evaluated using dedicated software (QMassMR v.8.1, Medis, Leiden, The Netherlands). Images will be assessed in the core lab by two experienced observers in CMR analysis, blinded to treatment allocation. Moreover, for checking consistency, each center will evaluate their own CMR images by a single blinded observer. Briefly, LV cardiac borders will be drawn in each cine image to obtain LV end-diastolic mass, LV end-diastolic volume (LVEDV), end-systolic volume (LVESV), and LVEF. LV mass and volumes normalized to the body surface area will be calculated using the modified Brody’s method^64^. The extent of edema and myocardial IS, both expressed as a percentage of LV mass, will be defined after manually tracing the endocardial and epicardial contours in T2WSTIR short-axis images and T1-IR-TFE short-axis images^65^, respectively. Both edema and myocardial IS will be identified as hyperintense regions, defined as >50% of the peak myocardial signal intensity (full width half maximum) with manual adjustment if required. When present, hypointense areas within the edematous or necrotic zone will be included in the edematous or necrotic region for quantification purposes. The presence of microvascular obstruction will be defined as hypointense areas within the hyperenhanced zone on late gadolinium enhancement images. Intramyocardial hemorrhage will be defined on T2W-STIR images as an hypointense core within the infarct hyperintense area.

#### Histopathology assessment

All animals in the subset B of experiments will be transferred to the catheterization laboratory for area-at-risk (AAR) assessment by Evans blue staining after 24 h of the procedure. The LAD will be reoccluded by angioplasty balloon inflation at the same level as the initial occlusion, following the same angiographic landmarks. Then, 100 mL of Evans blue dye (5%) will be infused into the LV cavity through a pigtail catheter while the balloon is inflated. Using this technique, nonischemic areas appear blue, whereas the AAR remains unstained^65^. The animals will be then euthanized with a lethal injection of sodium pentobarbital and their hearts will be subsequently excised. After 2 hours of freezing at −20 °C, the hearts will be sliced into six 8-mm section slices using a commercial rotator blade. These slides will be incubated in 2,3,5-triphenyltetrazolium chloride (TTC, 1% in saline) to demarcate the infarcted (white) versus viable (red) tissue^66^. All six slices will be then transferred into 10% neutral paraformaldehyde buffer for 20 min. Finally, high-resolution photographs will be taken using a digital camera and will be centrally assessed by the relevant core lab using ImageJ software v.1.45 s (National Institutes of Health, Bethesda, Maryland, United States). AAR will be expressed as a percentage of the total slice LV area (%) after manual delineation of cardiac contours. Myocardial infarct size will be assessed both as a percentage of the total slice LV area (primary endpoint) and as a percentage of the AAR (secondary endpoint).

#### Western blot analysis

Tissue sample prepared for Western blot analysis will be collected from ischemic, remote and border zone and thereafter snap-frozen in liquid nitrogen. The tissue will be then stored at −80 °C until further processing. The tissue will be homogenized in protein lysis buffer, containing Tris [50 nM], sacarose [320 Mm], NP40 0.2%, halt protease inhibitor cocktail (Sigma-Aldrich, P#8340) and two phosphatase inhibitor cocktails (Sigma-Aldrich, P#5726 and P#0044) and adjusted to pH 7. Small variations of the homogenization process will be allowed across centers. Homogenates will be then centrifuged for 15 min. at 13,000 g at 4 °C to remove debris and DNA. Protein content will be then determined using the Bradford assay and protein levels corrected accordingly to ensure equal protein loading. Between 30 and 50 µg of sample will be loaded on 10% acrylamide gels. Proteins will be electro-transferred onto PVDF 0.45 µm blotting membrane (Amershamm/Biorad) using wet transfer. The membranes will be blocked by incubating in 5% milk and subsequently incubated with appropriate primary antibodies at 4 °C during 18 h.

Primary antibodies to be used will be acquired from Cell Signaling Technology (Leiden, The Netherlands): Akt (#9272), Phospho-Akt (Ser473) (#4060), ERK1/2 (#9102), Phospho-ERK1/2 (Thr202/Tyr204) (#9101), Stat3 (#9139), Phospho-Stat3 (Tyr705) (#9145). Anti-GAPDH (Ambion, #AM4300) will be used as loading control. At 24 h, membranes will be probed with secondary infrared fluorescence antibodies. Protein levels will be quantified using ImageJ software v.1.45 s (National Institutes of Health, Bethesda, Maryland, United States).

#### Proteomics

Samples from the ischemic and remote myocardium from pigs allocated in the subset C of experiments will be collected within minutes of euthanasia and processed for proteomic analysis. Protein extracts will be obtained by tissue homogenization with ceramic beads (MagNa Lyser Green Beads apparatus, Roche, Germany) in extraction buffer (50 mM Tris-HCl, 1 mM EDTA, 4% SDS, pH 8.5). Free Cys residues will be blocked with 50 mM iodoacetamide at the time of protein extraction, following disulfide bonds Cys residues reduction with DDT (GE Healthcare) and alkylation with MMTS (Themo Scientific). Supernatant will be collected and protein concentration will be measured by RC/RD Protein Assay (BioRad). The protein extracts will be stored at −80 °C. The protein extracts will be stored at −80 °C. The protein extracts will be subjected to digestion overnight at 37 °C in FASP filters with trypsin (Promega, Madison, WI, USA) at a 40:1 protein:trypsin (w/w) ratio in 50 mM ammonium bicarbonate, pH 8.8. The resulting peptides will be desalted with C18 Oasis cartridges (Waters Corporation, Milford, MA, USA) using 50% acetonitrile (ACN) (v/v) in 0.1% trifluoroacetic acid (v/v) as eluent, and vacuum-dried. The peptides will be TMT-labeled following manufacturer’s instructions. A common pool of all samples will be used as an internal control for all TMT experiments. Labeled peptides from each TMT experiment were pooled, separated into 5 fractions by high-pH reverse phase peptide fractionation kit (Thermo Fisher Scientific), desalted and analyzed using an Easy nano-flow HPLC system (Thermo Fisher Scientific, Bremen, Germany) coupled via a nanoelectrospray ion source (Thermo Fisher Scientific) to a Q-Exactive mass spectrometer (Thermo Fisher Scientific). C18-based reverse phase separation will be used with a 2-cm trap column and a 50-cm analytical column (EASY column, Thermo Fisher Scientific) in a continuous acetonitrile gradient consisting of 0–30% A in 180 min, 50–90% B in 3 min (A = 0.1% formic acid; B = 90% acetonitrile, 0.1% formic acid), at a flow rate of 200 nl/min. Mass spectra will be acquired in a data-dependent manner, with an automatic switch between MS and MS/MS using a top 15 method. MS spectra in the Orbitrap analyzer will be analyzed in a mass range of 400–1500 m/z and 120,000 resolution. HCD fragmentation will be performed at 35 of normalized collision energy and MS/MS spectra will be analyzed at 30,000 resolution in the Orbitrap.

Database searches will be performed with Proteome Discoverer (version 2.1, Thermo Fisher Scientific) using SEQUEST (Thermo Fisher Scientific) against an Uniprot database containing pig and human sequences, as described^67^. Parameters will be selected as follows: trypsin digestion with 2 maximum missed cleavage sites, precursor mass tolerance of 800 ppm, fragment mass tolerance of 0.02 Da. Methionine oxidation (+15.994915) and cysteine carbamidomethylation of +57.021 Da and cysteine methylthiolation of +45.988 Da were set as variable modification. Lysine and peptide N-terminal modification of +229.162932 Da will be set as fixed modifications. The same collections of MS/MS spectra will also be searched against inverted databases constructed from the same target databases. Peptide identification from MS/MS data will be performed using the probability ratio method developed in our laboratory^68^. False discovery rates (FDR) of peptide identifications will be calculated using the refined method^69,70^; 1% FDR will be used as criterion for peptide identification. Each peptide will be assigned only to the best protein proposed by the Proteome Discoverer algorithm. For peptide quantification and statistical analysis, the quantitative information from TMT reporter intensities will be integrated from the spectrum level to the peptide level, and then to the protein level based on the WSPP model^71^ using the Generic Integration Algorithm (GIA)^72,73^. Protein abundance changes will be expressed in standardized units (${z}_{q}$). Oxidative modifications will be analyzed as described^74^.

### Randomization

Pigs will be centrally randomized via web (https://www.w3nexus.net/w3nexus/index_en.html). They will be allocated on a 1:1 ratio to either IPC or control arm using permuted block randomization, stratified by laboratory, to ensure the same number of pigs allocated to each arm in each center. In order to avoid selection bias, randomization will be done after the local center provides the identification of the animal and its weight. All randomized animals are included in the study, regardless of whether they complete the protocol or not in an intention-to-treat principle (see below).

### Data management and logistics

The unmeasured outcome of each set of experiment (images, pictures of tissue, samples for Western blot and proteomics) will be sent to the relevant core lab (CMR, histopathology, proteomics) or the relevant centers for Western blot analysis (3 laboratories). All these assessments will take place blinded to treatment allocation and raw data will be sent to the statistical core lab for their analysis. Due to the large amounts of data produced, Proteomics results will be firstly analyzed using specific algorithms by the core proteomics lab; the results will then be filtered and the quantitative values of a selected list of protein markers will then be sent to the statistical core lab.

### Sample size

Sample size was estimated for the primary endpoint, which is a reduction on myocardial IS measured by CMR, based on prior results observed by one of the research groups^59^. However, we assumed a less optimistic treatment effect given the expected heterogeneity (between-center variability on top of biological variability). Anticipating a mortality of up to 20% and considering a randomization ratio of 1:1, a sample size of 30 pigs (10 pigs per center; 5 pigs per arm in each center) are needed to detect a relative risk reduction of 50% (from 25% to 12.5%) with a power of 85% and an alpha error of 5%. This sample size should be enough for every single center to detect the expected treatment effect as well as to obtain an accurate overall treatment effect when pooling all the data and conducting a random-effect model in which some heterogeneity is allowed (between-experiments and between-center variation). This estimated sample size will be extended to the other two groups (subgroups B and C) under the assumption that the magnitude of the treatment effect will be the same across the remaining experimental endpoints.

### Statistical analysis plan

The primary statistical analyses will be conducted according to the intention-to-treat (ITT) principle. A secondary/sensitivity analysis will be performed on the per protocol (PP) set of pigs without considering potential crossovers or experimental failures. The null hypothesis is that myocardial IS is similar between IPC and control pigs, whereas the alternative two-sided hypothesis is that myocardial IS is reduced by 50% when measured by CMR.

Categorical data will be presented as absolute number and percentages and compared using either the chi-squared test or the Fisher’s exact test, as appropriate. Continuous variables will be expressed as mean ± standard deviation (SD) if normally distributed or as median and interquartile range (IQR) if not, and differences will be accordingly compared using the *t* test or the Mann–Whitney *U* test.

For each independent center, we will estimate the effect size (alongside its corresponding 95% CI) as a relative reduction on myocardial IS (risk ratio) and as an absolute reduction (raw difference in the percentage of myocardial IS) and will use linear regression models to establish comparisons between arms (IPC vs. control).

To estimate the overall effect with higher accuracy, all data relative to each set of experiments will be pooled and a random effect models, in which some within- and between-laboratories heterogeneity is allowed, will be used.

The degree of agreement between observers (either between the two CMR evaluators from the core lab or between them and each local evaluator) will be graphically evaluated through Bland-Altman plots as well as using the intraclass correlation coefficient (rI).

The robustness of our findings will be tested in a sensitivity analysis by performing an additional analysis using the standardized difference in means (SMD; the mean of the control group minus the mean of the IPC group, divided by the pooled SD of the two groups)^54,75^.

A similar statistical plan will be carried out for the secondary endpoints, with some variations according to the nature of the variables, except for proteomics results, which will require of other specific methods^67^.

The two-tailed significance level will be set at P < 0.05. STATA software version 13.1 (Stata Corp, College Station, TX, USA) and GraphPad Prism version 6.00 (GraphPad Software, La Jolla California, USA) will be used to perform the analyses and produce the graphs, respectively. The results will be reported according to the Animal Research: Reporting of *In Vivo* Experiments (ARRIVE) guidelines for reporting animal research^35^.

### Potential impact of the findings: CIBER-CLAP

In summary, there is sufficient evidence on controversial and inconsistent experimental findings supporting the need for a platform testing cardioprotective therapies in a multi-centric scale^5,11,25^. It is time to accept that the approach used in the last decades have failed to date to produce a therapy able to both provide IS-limiting effect and improve clinical outcomes^76^. Performing preclinical studies with the rigor of multicenter, RCTs is a paradigm shift in the field. Based on the principles of conducting standardized experimental protocols, randomization, blinding and assessment of reproducibility, this platform aims to assist in better identification of interventions with great potential to be translated into pilot clinical trials.

Unlike the CAESAR initiative^34^, our platform will involve more laboratories, thus adding further variability (external validity) and will imply a more comprehensive approach (CMR, histopathology, Western blot, proteomics). As a first step, our study will evaluate the consistency of the effect of IPC in a swine model of IRI across laboratories. This would be the basis to assess the reproducibility of further promising cardioprotective therapies. Using this platform, positive findings would be more solidly attributed to the cardioprotective intervention under investigation rather than potential biases or random error: reproducibility would play a central role in the decision-making of translating therapies from bench to bedside. From a methodological perspective, the publication of this study design is a breakthrough in the experimental field. Our study might be a turning point for the future of cardioprotection, experimental design and transparent reporting.

The EU-CARDIOPROTECTION COST Action (CA16225), a pan-European research network of leading experts in experimental and clinical cardioprotection, whose overall aim is to improve the translation of novel experimental cardioprotective therapies into the clinical setting for patient benefit will also contribute to the assessment of reproducibility in this field^77^. One of the main goals of this COST Action is to improve the pre-clinical evaluation of novel cardioprotective therapies by setting up a multicenter network of research centers capable of undertaking pre-clinical and clinical cardioprotection studies (termed the European Cardioprotection Consortium, ECC). The massive potential synergies between our CIBER-CLAP and the ECC should make the 2 networks to converge into the largest multicentre/multinational large animal platform. Conversations for such a collaboration have already been started.

### Limitations

Although in this study of IPC all core labs will be blinded to the results until the study will be completed, the nature of the IPC intervention makes impossible to blind local investigators to group allocation. In subsequent CIBER-CLAP studies, neither the researchers performing the experiments nor those at the core labs will have any knowledge on treatment assignment. Some factors might influence the treatment effect of IPC, such as differences in diet, animal size, breeding or the animal source (i.e from different vendors/farms). Although some anesthetic regimes have shown a cardioprotective effect, our protocol was chosen based on our previous successful experience protecting on top of using this anesthetic protocol with both metoprolol^51^ and ischemic conditioning^59^. Similarly, some techniques (i.e. CMR, Western Blot) might slightly differ across centres. However, we believe that allowing for few small variations across laboratories better reflect the reality and can potentially make our results further generalizable. The first randomized experimental preclinical executed by CIBER-CLAP assessing IPC do not evaluate the area of no-reflow by pathology standards (thioflavin staining). Although no-reflow will be evaluated by CMR, the lack of a pathology evaluation is a limitation. Subsequent studies of CIBER-CLAP will incorporate thioflavin staining as part of the protocol. Similarly, in the first trial assessing IPC, tissue sampling for the evaluation of signalling pathways has been set at 30 min reperfusion. Given that some protective signals are effected at 10 min reperfusion^48,49^, this protocol might miss some of these. Future CIBER-CLAP protocols will include an experimental arm where samples are collected 10 min after reperfusion.
